# Diffusion of clindamycin-resistant and erythromycin-resistant methicillin-susceptible *Staphylococcus aureus* (MSSA), potential ST398, in United States Veterans Health Administration Hospitals, 2003-2014

**DOI:** 10.1186/s13756-017-0212-1

**Published:** 2017-06-05

**Authors:** Margaret Carrel, Michihiko Goto, Marin L. Schweizer, Michael Z. David, Daniel Livorsi, Eli N. Perencevich

**Affiliations:** 1 Department of Geographical & Sustainability Sciences, University of Iowa, 305 Jessup Hall, Iowa City, IA 52242 USA; 2 Department of Epidemiology, University of Iowa, Iowa City, IA USA; 3 Iowa City VA Health Care System, Iowa City, IA USA; 4 Department of Internal Medicine, University of Iowa Carver College of Medicine, Iowa City, IA USA; 5 Department of Medicine, The University of Chicago Biological Sciences, Chicago, IL USA

**Keywords:** MSSA, ST398, Spatiotemporal analysis, Emergence, Diffusion

## Abstract

**Background:**

Changing phenotypic profiles of methicillin-susceptible *Staphylococcus aureus* (MSSA) isolates can indicate the emergence of novel sequence types (ST). The diffusion of MSSA ST can be tracked by combining established genotypic profiles with phenotypic surveillance data. ST398 emerged in New York City (NYC) and exhibits resistance to clindamycin and erythromycin but tetracycline susceptibility (“potential ST398”). Trends of potential ST398 were examined in a national cohort of all Veterans Health Administration patients with MSSA invasive infections during 2003–2014.

**Methods:**

A retrospective cohort of all patients with MSSA invasive infections, defined as a positive clinical culture from a sterile site, during 2003–2014 was created. Only isolates tested against clindamycin, erythromycin and tetracycline were included. Annual hospital-level proportions of potential ST398 were compared according to facility distance from NYC and region.

**Results:**

A total of 34,025 patient isolates from 136 VA medical centers met the inclusion criteria. Of those, 4582 (13.5%) met the definition of potential ST398. Potential ST398 increased over the 12-year cohort and diffused outwards from NYC. Incidence Rate Ratios of >1.0 (*p* < 0.01) reflect increases in potential ST398 over time in hospitals nearer to NYC.

**Conclusions:**

We observe an increase in the phenotypic profile of potential ST398 MSSA isolates in invasive infections in a national cohort of patients in the US. The increase is not evenly distributed across the US but appears to diffuse outwards from NYC. Novel MSSA strain emergence may have important clinical implications, particularly for the use of clindamycin for suspected *S. aureus* infections.

## Background

The emergence of novel genotypes of *Staphylococcus aureus* (SA), either methicillin-resistant (MRSA) or methicillin-sensitive (MSSA), has implications for the availability and efficacy of empiric therapy options. Detecting and tracking such an emergence is difficult, however, given the lack of nationwide genotyping databases. Isolates collected in routine clinical care are seldom genotyped and retained in an isolate bank for future analysis. As a result, analysis of phenotypic proxies for genotypes, based on susceptibility panels completed during routine clinical treatment, provide a method for hypothesis development regarding the emergence of novel strains. A change in SA susceptibility patterns would suggest changes in the underlying genotypic strains that are circulating and provide impetus for funding large-scale directed genotypic studies.

When genotyping studies for SA have been completed, they have overwhelmingly focused on MRSA [1]. Yet even in MRSA, epidemiological investigations of phenotypic susceptibility patterns have preceded in-depth genotypic studies [2–5]. For example, community-associated MRSA was initially recognized through distinct phenotypic patterns showing lack of resistance to certain antimicrobials [6–10]. MSSA receives less attention than does MRSA even though it is associated with similarly high rates of morbidity and mortality [11, 12].

A newly emerging strain that has been detected both phenotypically and genotypically in the US is sequence type 398 (ST398). ST398 MRSA is of human origin but is more often reported in the literature as having an association with livestock exposure [13–18]. ST398 MSSA has been observed in Belgium, China, France, the Netherlands, and Spain in individuals without livestock contact [19–23]. In the United States (US), ST398 MSSA was first observed in New York City (NYC) hospitals in 2004–2007, and was found predominantly in individuals who had connections with the Dominican Republic and who also reported no livestock contact [15, 24, 25]. ST398 MSSA was not observed in other studies with samples taken from across the US during this time [26, 27].

ST398 MSSA presents a case study for how phenotypic analysis can be used to document the potential diffusion over space and time of an emerging SA strain type. In systematic surveillance of NYC hospitals for MSSA ST398, Uhlemann et al. indicated that the majority of detected isolates shared a resistance profile: 100% (64/64) were tetracycline-susceptible and 97% (62/64) were clindamycin-resistant and erythromycin-resistant [24]. In contrast, only 38% of non-ST398 controls shared this profile. This susceptibility profile was observed in ST398 isolates in other studies [20, 22, 23, 28–30]. Using this profile as a proxy for ST398, Gandra et al. observed the early presence of potential ST398 in the state of New York followed by other northeastern locations and then regionally, findings in concordance with the timing of the emergence of this type of ST398 observed by Uhlemann et al. [31, 32].

The Veterans Health Administration (VHA) represents the only nationwide healthcare provider in the US, with standardized procedures for testing and reporting of antibiotic susceptibilities. This study makes use of this singular large national database to evaluate a possible emergent phenotypic profile over space and time. Using susceptibility profiles from patients with MSSA invasive clinical isolates, we sought to examine spatial and temporal trends in tetracycline-susceptible and clindamycin-resistant and erythromycin-resistant MSSA bacteria to determine whether this phenotype, which we designate as *potential ST398*, was present in and diffusing among veterans across the US. The emergence and diffusion of this novel MSSA phenotype, with resistance to frequently used antimicrobials, has important clinical implications for empiric therapy in US healthcare settings.

## Methods

A retrospective cohort of all patients from VHA facilities in the contiguous US with MSSA invasive infections during 2003–2014 was created. An invasive infection was defined as a positive clinical culture from a sterile body site such as blood or synovial fluid; specimens from non-sterile sources such as urine, sputum and wounds were excluded. Only those MSSA isolates that were tested against all three classes of antibiotics (tetracycline, lincosamides, macrolides) were included in the analysis. All microbiology laboratories within the VHA system are required to follow guidelines recommended by Clinical Laboratory Standards (including routine D-test to detect inducible clindamycin resistance for all SA isolates, effective 2004) [33]. The number of isolates in each hospital in each year that met the phenotypic profile for potential ST398 (lincosamide and macrolide resistant, tetracycline susceptible) was summed and the proportion of SA that was potential ST398 was calculated in two-year increments for each facility.

This study used medical, microbiological, and demographic data extracted from the Veterans Affairs’ (VA) electronic health record for the VA’s Corporate Data Warehouse and accessed through the VA Informatics and Computing Infrastructure, as previously described [34]. The institutional review board of the University of Iowa and the Research and Development Committee of the Iowa City VA Medical Center (VAMC) approved this study.

**Fig. 1:**
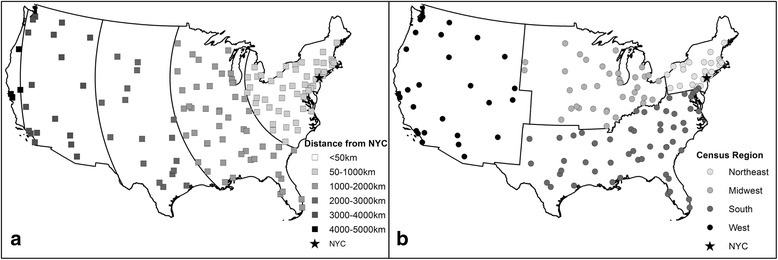
Location of VA Medical Centers (VAMCs) included in the study. Identified by **a** Distance from New York City and **b** Location within each Census Region

The distance of each VAMC from NYC was calculated in kilometers using ArcMap 10.2 (ESRI, Redlands, CA) (Fig. 1a). Facilities were further assigned to one of four Census regions (Northeast, South, Midwest, West), except for the three NYC VHA hospitals (two in Manhattan, one in the Bronx) which formed their own category, given the evidence for the emergence or early appearance of ST398 MSSA in NYC (Fig. 1b) [24, 31, 32].

A Poisson generalized estimating equation (GEE) regression model, with counts of potential ST398 in each VAMC in each two-year period as the outcome variable, explored the relationship between phenotypically potential ST398 and distance from NYC over time while accounting for repeat observations in hospitals over time. Because the majority of VAMCs had zero isolates matching the phenotypic profile in one or more years, an overdispersed model was fitted. Total invasive MSSA infections were included as an offset variable. Distance was included as a categorical variable, with hospitals grouped at <50 km from NYC and then in 1000 km increments. An interaction term between year and distance category was included in the model to detect regional variability in temporal trends, and incidence rate ratios (IRRs) for temporal trends for each distance category were calculated based on the model. Category-specific IRRs were also calculated for each distance category with hospitals >4000 km from NYC as the reference category to detect regional variability in baseline prevalence rates. Model diagnostics, including residual plots and dispersion measures, were examined to assess appropriateness of the model. Statistical analysis was completed in SAS version 9.4 (Cary, NC).

## Results

**Fig. 2:**
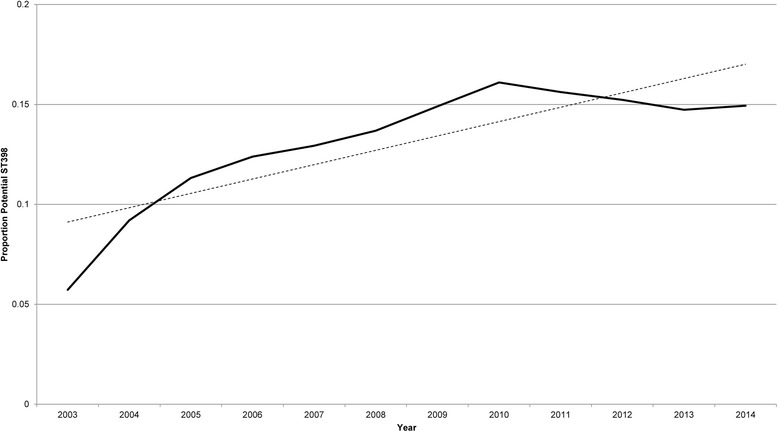
Proportion of isolates matching the potential ST398 susceptibility profile over time. *Solid line* represents observed data, *dotted line* is a fitted linear trend line

In 2003–2014, 47,513 patients from 136 VAMCs had sterile-site cultures positive for MSSA. Of these, 34,025 (72.2%) were tested against all three antibiotics. The number of sterile site MSSA isolates tested against all three antibiotics varied over time, rising from 62.2% in 2003 to 76.05% in 2014. Among the included sterile site MSSA isolates, 4582 (13.5%) exhibited clindamycin resistance and erythromycin resistance with tetracycline susceptibility and were potentially ST398. The proportion of isolates with this phenotypic profile increased over time, from a low of 5.7% in 2003 to a high of 16.1% in 2010, falling to 14.9% in 2014 (Fig. 2).

**Fig. 3:**
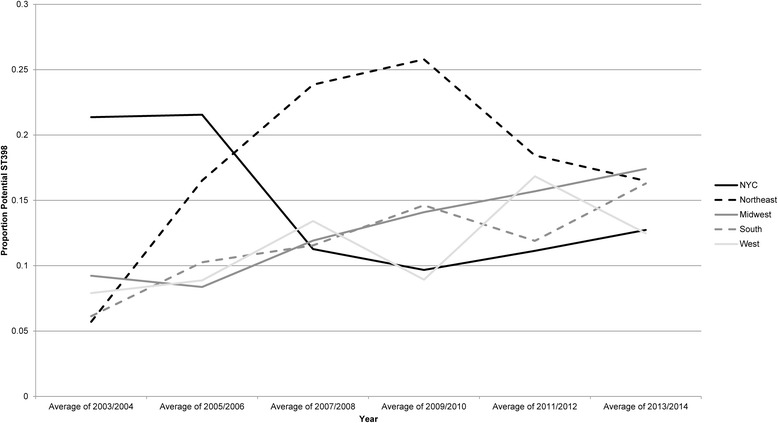
Proportion of potential ST398 MSSA in 2-year categories, stratified by Census region or NYC location

The proportion of isolates classified as potential ST398 based on their susceptibility profile was highest in the early years of the study (2003–2004) in NYC VAMCs (Fig. 3). Potential ST398 proportions then increased in Northeastern VHA facilities, remaining high through the middle years of the study period before falling in 2009–2010. Proportions of potential ST398 increased less sharply but were steadier over time in facilities in the Midwest, South and West.

**Fig. 4:**
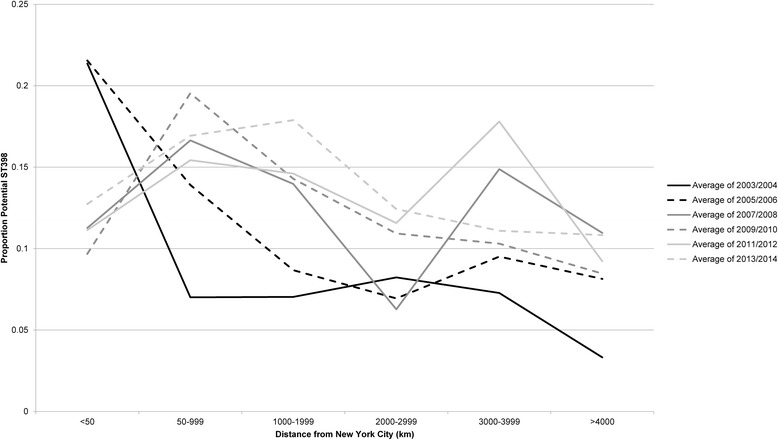
Potential ST398 proportions in VAMCs over time, stratified by distance from NYC

In charting potential ST398 versus distance from NYC, we observed that the proportion of isolates with a phenotypic profile of potential ST398 was lower in all distance categories in 2003–2004 than in subsequent years, concordant with the overall upward trend in potential ST398 observed in the entire cohort (Fig. 4). However, when facilities were stratified by distance from NYC rather than Census region, a distance decay effect was observed. In 2003–2004 and 2005–2006 the highest proportions of potential ST398 were observed in facilities closest to NYC; facilities at greater distances had less potential ST398. Peaks in potential ST398 were then observed in VHA hospitals at greater distances from NYC in 2007–2008 and 2009–2010. The overall increase in isolates with a potential ST398 genotype within the VHA system seen in Fig. 2 is further indicated in the higher proportions of potential ST398 seen in all distance categories in the later years of the study.

Results of the Poisson GEE regression indicated that, at the facility level, there was a significantly higher proportion of potential ST398 among MSSA isolates for hospitals in NYC (IRR = 1.81, *p*-value = 0.07) and the area closely surrounding NYC (50-999 km) (IRR = 1.74, *p*-value = 0.04) when compared to those facilities furthest from NYC (Table 1). Distance category-specific IRRs for annual trend indicated the proportion of potential ST398 significantly increased over time in facilities within 3999 km of NYC. There was statistically significant variability in temporal trends among distance categories (*p* < 0.01).

**Table 1:** Incidence rate ratios for isolates with clindamycin & erythromycin resistance and tetracycline susceptibility, potential ST398, in hospitals at distances of <4000 km from NYC versus potential ST398 in hospitals >4000 km of NYC and for interaction between distance and year

	IRR	95% CI	*p*-value
Distance <50 km	1.81	0.94–3.47	0.0741
Distance 50-999 km	1.74	1.01–2.96	0.0425
Distance 1000-1999 km	1.51	0.88–2.57	0.1283
Distance 2000-2999 km	0.96	0.53–1.72	0.8961
Distance 3000-3999 m	1.49	0.86–2.59	0.1518
Distance 4000-4999 km (reference)	1	--	--
Year*Distance <50 km	1.02	0.96–1.09	0.4878
Year*Distance 50-999 km	1.05	1.03–1.07	<0.0001
Year*Distance 1000-1999 km	1.06	1.04–1.08	<0.0001
Year*Distance 2000-2999 km	1.09	1.05–1.13	<0.0001
Year*Distance 3000-3999 m	1.04	1.01–1.06	0.0047
Year*Distance 4000-4999 km	1.07	0.99–1.16	0.0629

*indicates interaction

## Discussion

An almost three-fold increase in a novel phenotypic profile among MSSA isolates was observed in the VHA system between 2003 and 2014, though the increase did not occur evenly across the US. While the phenotypic definition used does not definitively indicate that these MSSA infections were caused by ST398, the spatiotemporal patterns observed in the emergence and diffusion of this resistance pattern are suggestive of ST398. Initially, we observed a rise in potential ST398 in NYC VHA facilities and lower proportions of potential ST398 in facilities further from NYC. However, this pattern changed over time, as potential ST398 diffused across the country, peaking in facilities in further distance categories in later years of this study. This pattern is consistent with previous reports of NYC as the origin of ST398 MSSA in the US and with the phenotypic pattern observed in non-VHA US hospitals [24, 31].

If ST398 MSSA is spreading within the VHA and more broadly in the US population, this is reason for concern. ST398 may be highly fit and virulent in both its MSSA and MRSA forms, and the reduction in treatment options associated with clindamycin resistance has important clinical implications. ST398 MSSA has been associated with efficient transmission between people, with greater capacity for adhesion to human skin [25]. The likely easy transmissibility of ST398 MSSA has previously been observed [30], and this could be why the prevalence of potential ST398 in the studied cohort increased over time. Because prior research has suggested that ST398 MSSA strains are associated with bloodstream infections and necrotizing pneumonia, this widespread diffusion and circulation within the US population has public health implications [17, 19]. The overall increase and geographic dispersion that we observed support the theory that ST398 is now circulating widely in the US. Even if the observed phenotypically-defined putative strain of MSSA is not ST398, it raises concern for a shift in the susceptibility of MSSA strains causing invasive infections in the VHA population of our national study.

Given the observed increase in resistance to erythromycin, the receipt of other macrolides, such as azithromycin, may be a key risk factor for acquisition and transmission of isolates with this phenotypic profile. Macrolides are frequently prescribed unnecessarily for acute respiratory tract infections due to viruses [35]. The association between macrolide use and ST398 infections could be tested in future studies. A prior study found that patients infected with ST398 were significantly more likely to have been hospitalized in the prior 6 months [24]. Therefore, it is possible that lapses in infection control within healthcare settings may be contributing to the spread of this strain.

Most clinical SA outcomes studies have focused on the role of methicillin resistance in driving increased mortality or treatment failure [36]. However, emergence of resistance to other clinically important antimicrobials, such as clindamycin, could have important implications for treatment selection and clinical outcomes. Clindamycin is associated with reduced recurrence rates and lower risk of treatment failure for SA and other types of skin & soft tissue infections (SSTIs) and is commonly used for the treatment of uncomplicated infections in pediatric populations [37–39]. The increasing prevalence of clindamycin resistance in MSSA isolates from clinically significant infections should prompt clinicians to re-evaluate this prescribing practice, particularly if local data indicate that clindamycin is not routinely active against SA isolates.

This study makes use of a proxy indication of ST398 genotype: the presence of resistance to clindamycin and erythromycin but susceptibility to tetracycline. Genotyping of the approximately 34,000 MSSA isolates between 2003 and 2014 was not standard practice, nor economically practical, within the VHA system. VHA data, like most other MSSA data, is collected for clinical rather than research purposes, so use of a phenotypic proxy was necessary. It is possible that a MSSA isolate exhibits the phenotypic profile associated in other studies with ST398 but is, in fact, another type altogether, as has been previously observed, in ranges of 19–38% in some studies [22–24, 31]. Alternately, some ST398 MSSA do not share this profile [24]. Use of a non-definitive profile could lead to a large overestimation of the presence of potential ST398 MSSA within the VHA, though this phenotypic proxy has been previously utilized for nationwide surveillance [31].

Only MSSA infections considered invasive were studied in this analysis. Isolates from non-sterile sites were not included. This potentially created selection bias in the cohort in that only relatively severe MSSA cases were included. Another limitation is that all facilities were not testing MSSA isolates against clindamycin, erythromycin and tetracycline in all years. Although the number of isolates tested against all three antibiotic classes rose during the study period, a little over one-quarter of MSSA isolates were not tested against all three and were excluded from analysis. The variation in assessment of resistance across time may result in an underestimate of the number of isolates meeting the ST398 profile and could mask actual patterns of prevalence and diffusion of potential ST398 MSSA across the US. However, we expect resistance testing over time to be non-differential or random with regards to underlying phenotypic profile, and thus it should have little impact on our reported findings. An additional source of uncertainty is that while all VHA laboratories are required to adhere to CLSI (formerly NCCLS) guidelines, such as for testing inducible clindamycin resistance, we do not have information on adherence in individual facilities. Finally, the VHA patient population, comprised of predominantly older white males, is not representative of the US population as a whole.

We cannot definitively conclude the observed change in phenotypic profiles from 2003 to 2014 in the VHA MSSA cohort is due solely to ST398. However, there is circumstantial evidence that ST398 MSSA is emerging based on similarities to genotyped isolates in the literature and a similar association with distance from NYC, the putative epicenter of ST398 MSSA in the US. Within the VHA data, we document higher prevalence of isolates with a specific phenotypic profile in NYC hospitals in 2003–2004 with lower prevalence at greater distances from NYC. This pattern changed over the years of the study as overall potential ST398 increased in facilities further from NYC.

In contrast to MRSA, seldom are nationally representative sets of MSSA isolates stored and genotyped. Until a nationwide data bank of isolates becomes available for genotypic evaluation, phenotypic surveillance of novel type emergence, followed by genotypic analysis in local healthcare settings, will have to suffice. Such phenotypically informed and directed genotypic analysis is more efficient and cost-effective than efforts to genotype all clinical isolates.

## Conclusions

Based upon a retrospective analysis of MSSA susceptibility data, our findings document the emergence and diffusion of a changing MSSA susceptibility pattern, suggestive of potential ST398, which occurred within the VHA system between 2003 and 2014. The diffusion of a novel MSSA genotype that is resistant to commonly-prescribed antimicrobials, and that is potentially more fit and transmissible than other genotypes, warrants attention as it can limit the options for empiric antimicrobial therapy in patients with suspected SA infections. Phenotypic profiles generated during routine clinical care can be used to evaluate hypotheses about changing genotypic patterns and to target isolates for detailed genotyping by standard methods such as spa typing, pulsed-field gel electrophoresis (PFGE) or even whole genome sequencing. Finally, these findings also suggest that establishing a nationwide surveillance system with banked isolates, regardless of their resistance to methicillin and other beta-lactams, could have significant epidemiologic and clinical utility.

## Abbreviations

GEE: Generalized Estimating Equation
MRSA: Methicillin resistant *Staphylococcus aureus*
MSSA: Methicillin sensitive *Staphylococcus aureus*
NYC: New York City
PFGE: pulsed-field gel electrophoresis
SA: *Staphylococcus aureus*
US: United States
VAMC: Veterans Affairs Medical Center
VHA: Veterans Health Administration
